# Ciliary Neurotrophic Factor Promotes the Migration of Corneal Epithelial Stem/progenitor Cells by Up-regulation of MMPs through the Phosphorylation of Akt

**DOI:** 10.1038/srep25870

**Published:** 2016-05-13

**Authors:** Jialin Chen, Peng Chen, Ludvig J. Backman, Qingjun Zhou, Patrik Danielson

**Affiliations:** 1 Department of Integrative Medical Biology, Anatomy, Umeå University, Umeå, Sweden; 2 State Key Laboratory Cultivation Base, Shandong Provincial Key Laboratory of Ophthalmology, Shandong Eye Institute, Shandong Academy of Medical Sciences, Qingdao, China; 3 Department of Clinical Sciences, Ophthalmology, Umeå University, Umeå, Sweden

## Abstract

The migration of limbal epithelial stem cells is important for the homeostasis and regeneration of corneal epithelium. Ciliary neurotrophic factor (CNTF) has been found to promote corneal epithelial wound healing by activating corneal epithelial stem/progenitor cells. However, the possible effect of CNTF on the migration of corneal epithelial stem/progenitor cells is not clear. This study found the expression of CNTF in mouse corneal epithelial stem/progenitor cells (TKE2) to be up-regulated after injury, on both gene and protein level. CNTF promoted migration of TKE2 in a dose-dependent manner and the peak was seen at 10 ng/ml. The phosphorylation level of Akt (*p*-Akt), and the expression of MMP3 and MMP14, were up-regulated after CNTF treatment both *in vitro* and *in vivo*. Akt and MMP3 inhibitor treatment delayed the migration effect by CNTF. Finally, a decreased expression of MMP3 and MMP14 was observed when Akt inhibitor was applied both *in vitro* and *in vivo*. This study provides new insights into the role of CNTF on the migration of corneal epithelial stem/progenitor cells and its inherent mechanism of Up-regulation of matrix metalloproteinases through the Akt signalling pathway.

The cornea plays an important role in the visual system. It protects the eye from outside insults and infections, and maintains the normal function of the eye by refracting the light. Cornea epithelium is located at the anterior corneal surface and has a stratified squamous structure. The limbal stem cells (LSCs) are located in the basal limbal epithelium, which is thought to be important for the corneal epithelium replenishment through “XYZ hypothesis”; in which X refers to the proliferation of basal limbal epithelial cells, Y to the migration of transient amplifying cells (TACs) towards the centre of cornea, and Z to the desquamation of terminally differentiated cells^1^. This limbal theory has recently been strongly supported by the lineage tracing of cytokeratin 14 positive (K14+) LSCs, which has demonstrated the centripetal migration process directly by multicolour^2^,^3^. The migration of LSCs is not only an important step for the normal homeostasis of corneal epithelium, but also for the corneal epithelial regeneration. It has been found that upon corneal wounding, cells migrate from the limbus and contribute to the corneal repair^4^. Moreover, patients with loss of LSCs, or dysfunction thereof, often suffer from corneal neovascularization, loss of corneal transparency, and visual impairment^5^,^6^,^7^.

Ciliary neurotrophic factor (CNTF) is one of the neurotrophic factors belonging to the IL-6 family. It is known that the Jak-STAT pathway is involved in CNTF signalling, in which CNTF binds to its receptor and forms a trimeric receptor together with leukemia inhibitory factor (LIF)-receptor subunit and gp130. Subsequently, Jak 1/2 is activated and STAT1 and/or STAT3 is phosphorylated and translocated into the nucleus to initiate gene transcription^8^. As a neurotrophic cytokine, CNTF has been shown to regulate the neuron survival, neurogenesis, differentiation of retinal stem cells, and nerve regeneration^9^,^10^,^11^,^12^. The cornea is one of the most densely innervated tissues in the body. It has recently been reported that CNTF promotes corneal epithelial wound healing through the activation of STAT3 in corneal epithelial stem/progenitor cells^13^. Furthermore, Akt (Protein kinase B) signalling was also activated after CNTF treatment^13^. However, the biological effect of activated Akt has not been clarified.

CNTF has been shown to stimulate migration in various cell types, such as endogenous progenitors during myelin repair^14^ and neuronal migration *in vitro*^15^. The expression of CNTF has not been detected in normal human cornea by immunofluorescence^16^. However, it has been shown to be elevated in oxidative stressed corneal endothelium^17^, and in diseases such as in pterygeal tissues^18^ and keratoconus (KC) cornea^19^. Even so, CNTF expression in the cornea after injury has yet not been reported, and it is furthermore unknown whether CNTF promotes the migration of corneal epithelial stem/progenitor cells during the repair process. In the present study, we show that mouse corneal epithelial stem/progenitor cell migration is promoted after CNTF treatment, and that this is mediated by matrix metalloproteinases (MMPs) through Akt signalling activation.

## Results

### Injury causes up-regulated expression of CNTF

**Figure 1:**
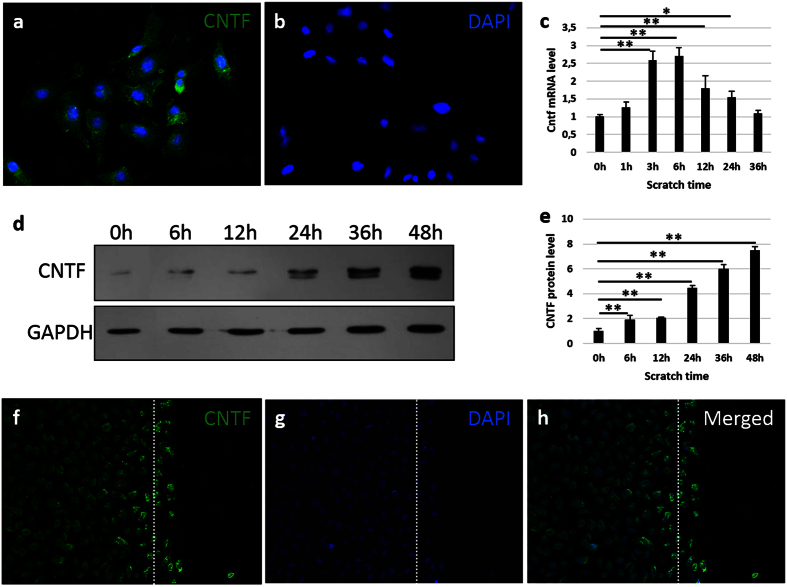
The expression of CNTF is up-regulated after injury. The cultured TKE2 cells were stained with CNTF (**a**), compared to negative control (**b**). (**c**) Gene expression of *Cntf* at post-injury 0 h, 1 h, 3 h, 6 h, 12 h, 24 h and 36 h was compared. The levels of *Cntf* are normalized to the 0 h time point. ‘h’ indicates hours. (**d,e**) Protein was extracted and evaluated at 0 h, 6 h, 12 h, 24 h, 36 h and 48 h after scratch. The levels of CNTF are normalized to the 0h time point. ‘h’ indicates hours. (**f–h**) The injury area of cultured cells was stained with CNTF at 16 h post scratching. h is the merged pictures of (**f**,**g**). Dash line indicates the edge of scratch caused by the tip. n = 3 per group. The experiments were performed three times with similar results. Statistical analysis was performed using One-way ANOVA and Bonferroni post hoc test. *Significant difference between two time-points at p < 0.05; **Significant difference between two time-points at p < 0.01.

The expression of CNTF was observed by immunofluorescent staining in the mouse corneal epithelial stem/progenitor cell line (TKE2) (Fig. 1a). After the scratch injury, the gene expression of *Cntf* increased after 1 hour, and reached a peak after 6 hours by approximately 3-fold as compared to 0 hour (Fig. 1c). The protein level of CNTF increased from 6 hours, and reached approximately an 8-fold increase at 48 hours (Fig. 1d,e). Notably, the cells within the scratch injury area showed higher expression of CNTF after 16 hours, as compared to the non-injured area (Fig. 1f–h).

### CNTF promotes the migration of mouse corneal epithelial stem/progenitor cells

**Figure 2:**
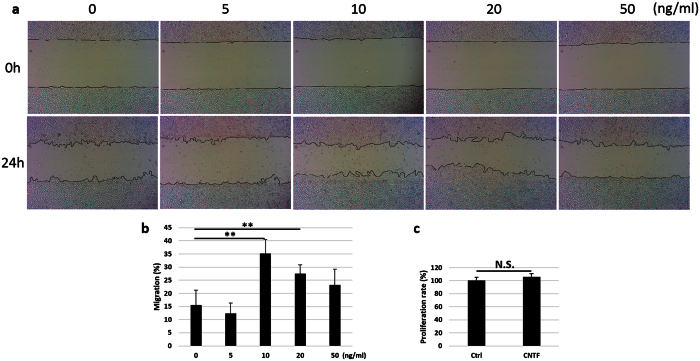
CNTF promotes migration of mouse corneal epithelial stem/progenitor cells. (**a**) Mouse TKE2 cells were incubated with 0, 5, 10, 20 and 50 ng/ml CNTF for 24 hours after the scratch was performed. Pictures at 0 hour and 24 hour were captured. (**b**) Image-Pro Plus 6.0 quantified the gap area and the migration percent was calculated. n ≥ 3 per group. The experiments were performed three times with similar results. Statistical analysis was performed using One-way ANOVA and Bonferroni post hoc test. **Significant difference between two groups at p < 0.01. (**c**) The proliferation rate was measured between the control and 10 ng/ml CNTF treated group at 24 hour after injury. ‘Ctrl’ indicates control group. n = 5 per group. Statistical analysis was performed using Student’s t-test. ^N.S.^No significant difference between two groups at p ≥ 0.05.

To evaluate the effect of CNTF on the migration of TKE2 cells, dose-dependent experiments was carried out. Different concentration of CNTF (0, 5, 10, 20, 50 ng/ml) was used to treat the cultured cells for 24 hours following the scratch injury (Fig. 2a). The migration percent peaked at 35.2 ± 5.3% in the 10 ng/ml CNTF treated group, as compared to 15.5 ± 5.6% in the control group (Fig. 2b). This promotion effect decreased gradually at higher concentration, i.e. 20 and 50 ng/ml (Fig. 2b). There was no difference in the cell proliferation and apoptosis after 24 hours in cells treatment with 10 ng/ml CNTF as compared to control (Fig. 2c and Supplementary Fig. 1), which excludes the possible interference of proliferation on cell migration at this time point.

### Akt and matrix metalloproteinases mediate the migration caused by CNTF

**Figure 3:**
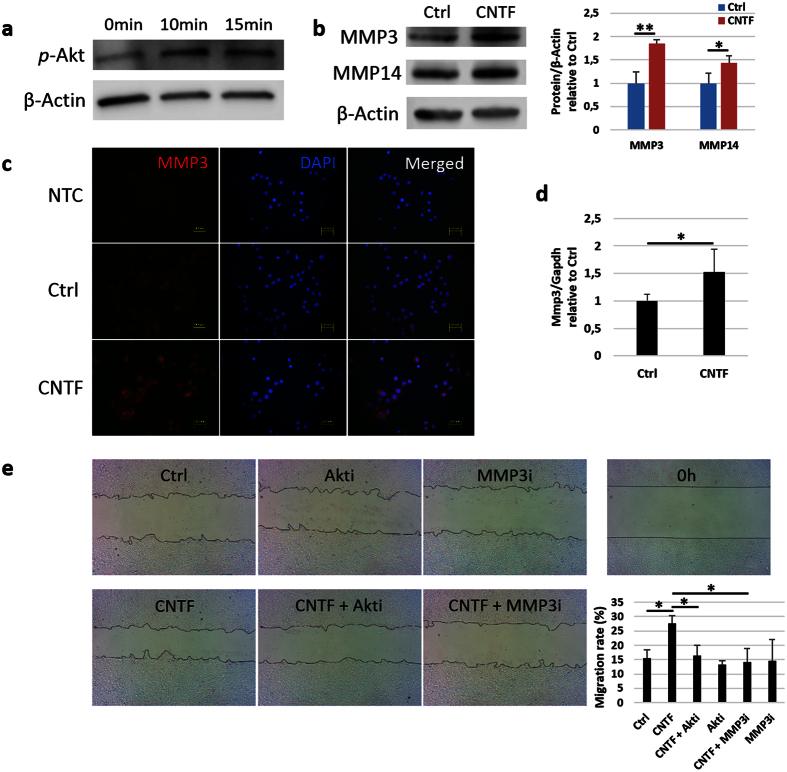
Akt and MMPs mediate the migration caused by CNTF. (**a–d**) Mouse TKE2 cells were treated with 10 ng/ml CNTF. (**a**) The expression of *p*-Akt was analysed by Western blot. (**b**) The expression of MMP3 and MMP14 was analysed by Western blot and quantified relative to β-Actin. (**c**) Immunofluorescence staining was performed to analyse the expression of MMP3. NTC, negative control; Ctrl, control group; CNTF, CNTF treated group. The right column is the merged picture of left column and middle column. (**d**) qPCR was performed to analyse the expression of *Mmp3*. (**e**) Confluent monolayer of TKE2 cells was scraped and treated with 10 ng/ml CNTF or not for 24 hours. For Akt or MMP3 inhibition, cultured TKE2 cells were pretreated with 40 μM Akt inhibitor for 30 minutes before CNTF treatment, or treated with 10 ng/ml CNTF simultaneously with 5 μM MMP3 inhibitor, for 24 hours. Cells treated with only Akt inhibitor or MMP3 inhibitor were also used as control. Image-Pro Plus 6.0 quantified the gap area at 0 hour and 24 hour and the migration percent was calculated. Akti, Akt inhibitor; MMP3i, MMP3 inhibitor. n = 3 per group. The experiments were performed three times with similar results. (**b,d**) Statistical analysis was performed using Student’s t-test. (**e**) Statistical analysis was performed using One-way ANOVA and Bonferroni post hoc test. *Significant difference between two groups at p < 0.05. **Significant difference between two groups at p < 0.01.

The expression of *p*-Akt was up-regulated after TKE2 cells were treated with 10 ng/ml CNTF for 10 and 15 minutes (Fig. 3a). Western blot experiments showed that CNTF induced the expression of MMP3 protein by 1.8-fold and MMP14 protein by 1.4-fold (Fig. 3b). Stronger fluorescence of MMP3 was observed in the CNTF group as compared to control (Fig. 3c). The *Mmp3* gene expression was up-regulated by 1.5-fold after 24 hours of treatment with CNTF (Fig. 3d).

To prove the mediation role of *p*-Akt and MMPs on cell migration caused by CNTF, Akt or MMP3 inhibitor was used to treat TKE2 cells in the scratch injury model in the presence of CNTF or not (Fig. 3e). The migration rate decreased by 28% when cells were treated with Akt inhibitor together with CNTF as compared to CNTF alone, and the MMP3 inhibitor together with CNTF decreased the migration by 31% as compared to CNTF alone. There was no significant difference between control group and inhibitors alone treatment.

### Akt regulates MMP3 and MMP14 in mouse corneal epithelial stem/progenitor cells

**Figure 4:**
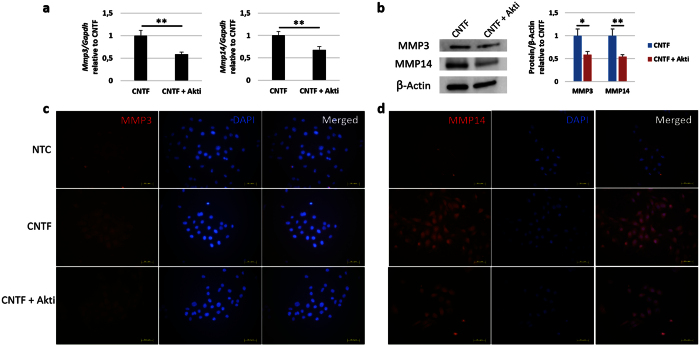
Akt regulates the expression of MMP3 and MMP14 in mouse corneal epithelial stem/progenitor cells. (**a**) The mRNA level of *Mmp3* and *Mmp14* was evaluated by qPCR after Akt inhibitor treatment together with CNTF as compared to CNTF alone. Akti, Akt inhibitor treatment. (**b**) The protein level of MMP3 and MMP14 was evaluated by Western blot. (**c,d**) The expression of MMP3 and MMP14 after Akt inhibitor treatment was evaluated by immunofluorescence staining. The right column is the merged picture of left column and middle column. n = 3 per group. The experiments were performed three times with similar results. Statistical analysis was performed using Student’s t-test. *Significant difference between two groups at p < 0.05. **Significant difference between two groups at p < 0.01.

To see if Akt regulated the expression of MMP3 and MMP14 in migration, cells treated with CNTF or CNTF together with the Akt inhibitor were collected and analysed on the level of gene and protein. It was seen that *Mmp3* gene expression was decrease by 42% in the Akt inhibitor group as compared to CNTF alone (Fig. 4a). The Immunofluorescence results confirmed this in the protein level (Fig. 4c). In Western blot quantification it was seen that the MMP3 protein level was decrease by 41% in the Akt inhibitor group as compared to CNTF alone (Fig. 4b). For MMP14, 32% and 45% decrease in expression was found in the gene (Fig. 4a) and protein level (Fig. 4b,d), respectively, after the treatment of Akt inhibitor as compared to CNTF alone.

### Akt contributes to the elevation of MMP3 and MMP14 *in vivo*

**Figure 5:**
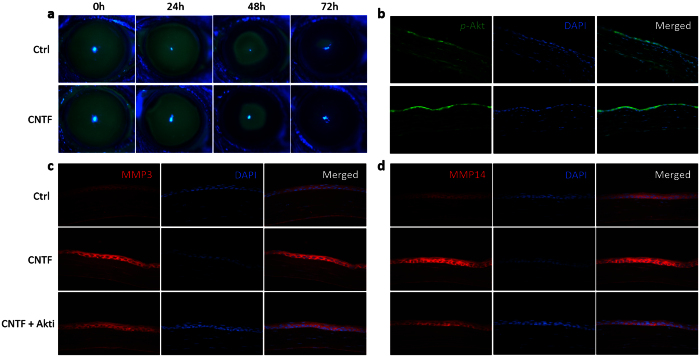
Akt contributes to the elevation of MMP3 and MMP14 *in vivo*. Normal mice were injected subconjunctivally with 50 ng CNTF or PBS (control group) after the scrape of corneal epithelium. Six mice were used in each group (n = 6). Immunofluorescence intensity of *p*-Akt, MMP3 and MMP14 was compared between groups in the regenerated region of corneal epithelium at 48 hour post scratching. Representative figures are shown. (**a**) The defect area of corneal epithelium was evaluated after 24, 48 and 72 hours by 0.25% fluorescein sodium under a BQ900 slit lamp. ‘h’ indicates hours. (**b**) Immunofluorescence staining was carried out to compare the expression of *p*-Akt. The right column is the merged picture of left column and middle column. (**c**,**d**) For Akt inhibition, the mice were injected with the Akt inhibitor 24 hours before CNTF injection. Immunofluorescence staining was carried out to compare the expression of MMP3 (**c**) and MMP14 (**d**) between groups.

In the results of animal experiments, CNTF accelerated the repair of corneal epithelial wound. The defect area was smaller in the CNTF treated group as compared to the control group at 24, 48, and 72 hours after the scrape of corneal epithelium (Fig. 5a). By Immunofluorescence, stronger staining intensity of *p*-Akt, MMP3 and MMP14 was observed in the regenerated region of corneal epithelium after 48 hours treatment with CNTF (Fig. 5b–d). The staining intensity of *p*-Akt, MMP3 and MMP14 was less evident in the region of the corneal stroma, as compared to in the regenerated region of corneal epithelium. Akt inhibitor was injected subconjunctivally before the application of CNTF and the scrape of corneal epithelium. It was found that Akt inhibitor treatment attenuated the promotion of CNTF on the elevation of MMP3 and MMP14 (Fig. 5c,d). Moreover, the regulation effect of Akt on the expression of MMP3 and MMP14 was also found in corneal epithelium scraped mice in the absence of CNTF (Supplementary Fig. 2).

## Discussion

This study elucidates the cell migration effect of CNTF in corneal epithelial stem/progenitor cells, and its inherent signalling mechanism through Akt and MMPs. CNTF promoted TKE2 cell migration in a dose-dependent manner *in vitro* and accelerated the corneal epithelial wound healing *in vivo*. Treatment with CNTF induced the expression of Akt phosphorylation (*p*-Akt), MMP3, and MMP14 both *in vitro* and *in vivo*. The effect of CNTF on cell migration was decrease by treatment of Akt and MMP3 inhibitors. Moreover, Akt inhibitor treatment reduced the expression of MMP3 and MMP14, both on gene and protein level *in vitro* as well as *in vivo*. The results of this study suggest a new role of CNTF in corneal epithelial stem/progenitor cells, which supports a regeneration-promoting effect of CNTF in corneal epithelium.

CNTF is one of the neurotrophic factors that have been found in diseased corneas^18^,^19^. Neurotrophic factors are a family of polypeptides with similar function in protecting the survival of the neurons, mainly including neurotrophins, glial cell-line derived neurotrophic factor family ligands (GFLs), and neuropoietic cytokines^20^. Neurotrophic factors are secreted by abundant nerve fibres, which are important for the maintenance and repair of cornea^21^. Degenerative neurotrophic keratitis could be formed if the neurotrophic system is disrupted, with decreased corneal sensitivity and repair ability^22^,^23^. It has recently been reported that CNTF promotes corneal epithelial healing by activating corneal epithelial stem/progenitor cells^13^. The expression of stem cells markers, such as ΔNP63 and TCF4, are regulated by STAT3 after CNTF treatment. The phosphorylation level of Akt is also elevated by CNTF, but do not contribute to the activation of limbal stem cells^13^. However, the activation of Akt signalling is known to be important for corneal epithelial healing, which is down-regulated in diabetes^24^,^25^. The involvement of Akt activation has been reported in the promotion of diabetic corneal epithelial healing by the neuropeptide substance P (SP)^26^ and by TGFβ3^27^. Our current study explored the role of *p*-Akt after CNTF treatment in the migration of corneal epithelial stem/progenitor cells, and it turned out that it is of great importance to the effect of CNTF in cornea epithelial regeneration.

The phosphorylation level of STAT3 is elevated both *in vitro* and *in vivo* after CNTF treatment^13^. We have found that a STAT3 inhibitor reduces the migration effect of CNTF on TKE2 cells (Supplementary Fig. 3a,b). However, the expression of MMP3 and MMP14 was not decreased after the treatment of STAT3 inhibitor (Supplementary Fig. 3c–f), which indicates that STAT3 signalling in itself participates in the cell migration process, but through other signalling mechanisms than MMP3 and MMP14. Further work needs to be performed to elucidate the mechanism of STAT3 in the migration of corneal epithelial stem/progenitor cells.

The MMP family contains a group of extracellular enzymes that have been widely reported in extracellular matrix (ECM) degradation, which is involved in various would healing processes, such as in skin and cornea^28^. The main functions of MMPs in corneal wound healing are to facilitate the keratocytes to repopulate the wound area and to assist in re-epithelialization^29^,^30^, which is related to cell proliferation and migration^30^. It has been found that there is increased gene expression of *Mmp1*, *Mmp9*, *Mmp10*, *Mmp12* and *Mmp13* in the mice corneal epithelium after corneal abrasion injury^31^, and the expression of *Mmp1*, *Mmp3*, *Mmp7* and *Mmp12* were increased in rat corneal epithelial cells during Wnt 7a-induced wound healing^32^. Nevertheless, the role of MMPs in epithelial migration has only been clearly demonstrated for a few members of the MMP family, especially concerning epithelial migration in the cornea^33^. The role of MMP9 in corneal epithelial cell migration has been mostly investigated^28^,^34^,^35^. However, in our preliminary study, the gene expression of *Mmp9* was detected in a very low level in the TKE2 cells both with and without CNTF treatment. The similar phenomenon was observed in *Mmp13* gene expression, which has been found to be involved in the migration of keratinocytes^36^. There are many reports about MMP14 in the invasion of different cancers, such as hepatocellular carcinoma^37^, tongue squamous cell carcinoma^38^, and lung cancer^39^. It is noticed that MMP14 could activate other MMPs, including MMP9^28^. However, the role of MMP14 in corneal epithelial cell migration is not known. Also, the effect of MMP3 in epithelial migration is poorly defined. Recently, Gao and colleagues showed that the migration of corneal epithelial cells is enhanced by the combination of topographically patterned substrates and electric fields, in a MMP3-dependent manner^40^, which indicates the possible importance of MMP3 in epithelial migration. In the present study, the expression of MMP3 and MMP14 were up-regulated after CNTF treatment both *in vitro* and *in vivo*, and their expression was decreased by Akt inhibitor treatment. Furthermore, the MMP3 inhibitor blocked the migration effect caused by CNTF. Since a specific inhibitor for MMP14 was not used in this study, it does not say whether inhibition of MMP14 specifically would decrease the migration effect of CNTF. However, based on the current data, it is clear that CNTF promotes corneal epithelial stem/progenitor cell migration at least partially through MMP3.

It is considered, that decreased corneal nerve fibre density after corneal injury contributes to the delayed repair effect because of the loss of trophic influences^22^,^23^. Some studies have reported the spatial localization of different neurotrophic factors in cornea^16^,^19^. However, the biological function demonstrated in animal studies or human clinics and its inherent mechanism have not been well elucidated. Nerve growth factor (NGF) is a well-known neurotrophic factor, which was discovered half a century ago and has been studied intensively since then. It has been reported that NGF treatment could promote the repair of the cornea, and restore its integrity, in patients who suffer from neurotrophic corneal ulcers^41^. The effect of CNTF in cornea epithelial repair and its mechanism are demonstrated by the present study combined with the previous one^13^. As the structure similarity and the regulation effect of neurotrophic factors in neurons and other cell types are similar, it is worth to study the roles of other neurotrophic factors in the physiological and pathological conditions of cornea, and their potential for future applications in clinic.

In summary, the current study explored a new role of CNTF in promoting cell migration of corneal epithelial stem/progenitor cells *in vitro* and repair of corneal epithelial wound *in vivo*. The migration was promoted by CNTF in a dose-dependent manner through an Akt and MMP signalling mechanism, since the effect of CNTF on cell migration was decrease by treatment of Akt and MMP3 inhibitors. Moreover, Akt inhibitor treatment reduced the expression of MMP3 and MMP14, on both gene and protein level, *in vitro* as well as *in vivo.* This study provides new insights into the mechanisms of CNTF on the migration of corneal epithelial stem/progenitor cells and the repair of corneal epithelium.

## Methods

### Cell culture

Mouse corneal epithelial stem/progenitor cell line (TKE2) was purchased from Public Health England (Cat No: 11033107), and cultured in keratinocyte serum-free medium (KSFM, Life technologies, Grand Island, NY, USA) supplemented with bovine pituitary extract (BPE) and epidermal growth factor (EGF) according to manufacturer’s instructions. This cell line^42^ was confirmed for positive expression of p63α, p40, K15, K17, ABCG2, TCF4 and Bmi-1, and for negative expression of K12. Cells were starved overnight in fresh KSFM (without BPE and EGF) before the treatment of CNTF (R&D Systems, San Diego, CA). The cultured cells were treated with 10 ng/ml CNTF and inhibitors for the measurement of *p*-Akt, MMP3 and MMP14 in mRNA accumulation and protein expression.

For Akt, STAT3 and MMP3 inhibition, cultured TKE2 cells were pretreated with 40 μM Akt inhibitor (Calbiochem, #124012, La Jolla, CA) or 100 μM STAT3 inhibitor (Calbiochem, #573096) for 30 min before CNTF treatment, or treated with 10 ng/ml CNTF simultaneously with 5 μM MMP3 inhibitor (Calbiochem, #444218) for 24 hours.

### Evaluation of CNTF expression after scratch and cell migration assay

Cell migration experiments were carried out in 6 well plates by doing a scratch in a confluent monolayer of TKE2 cells using a 1ml tip in the centre of the culture dish. To evaluate the expression of CNTF after injury, samples were collected for qPCR and Western blot analysis at different time-point after the scratch.

The effects of CNTF and certain inhibitors on cell migration were evaluated. After the scratch, cells were cultured with KSFM medium supplemented with different concentration of CNTF (0, 5, 10, 20, 50 ng/ml) for 24 hours. The inhibitors were used as mentioned above. Pictures at 0 hour and 24 hours were captured. Image-Pro Plus 6.0 quantified the gap area and the migration percent was calculated.

### Cell proliferation assay

Cell proliferation was measured using the CellTiter 96^®^ AQ_ueous_ One Solution Cell Proliferation Assay (Promega, Fitchburg, WI, USA). Cells cultured for 24 hours with or without the treatment with 10 ng/ml of CNTF were incubated in CellTiter 96^®^ AQ_ueous_ One Solution Reagent in a 5% CO_2_ incubator at 37 °C for 1 hour. The absorbance of the culture medium was measured at 490 nm. The data was shown relative to the control group, which was cultured for 24 hours without CNTF treatment.

### qPCR

Total RNA was extracted from cultured cells using RNeasy^®^ Mini Kit (Qiagen, Venlo, The Netherlands). cDNA were synthesized using High Capacity cDNA Reverse Transcription Kit (Applied Biosystems, Carlsbad, USA). qPCR was carried out using SYBR^®^ Green reagents and the ViiA™ 7 Real-Time PCR System (Applied Biosystems). All primers (Invitrogen, Carlsbad, CA, USA; Table 1) were designed using primer 5.0 software. Representative results are displayed as target gene expression normalized to Glyceraldehyde 3-phosphate dehydrogenase (*Gapdh*).

### Western blot analysis

Cultured cell samples were lysed in RIPA buffer. Protein was extracted and quantified using Bio-Rad Protein Assay (Bio-Rad, Hercules, CA, USA). Protein samples were loaded into pre-cast polyacrylamide gel (Bio-Rad) and ran for 1 hour at 150 V and later transferred to a PVDF Blotting Membrane (GE Healthcare, Little Chalfont, Buckinghamshire, UK) for 1 hour at 100 V. The blots were blocked in 5% bovine serum albumin (BSA) in TBST for 1 hour, and incubated with primary antibodies (Table 2) at 4 °C overnight. After washed with TBST for three times, the blots were incubated with a HRP-linked secondary antibody (Table 2) for 1 hour. After an additional wash in TBST for three times, the blots were incubated with ECL (GE Healthcare) to visualize the bands using Odyssey^®^ Fc Dual-Mode Imaging System (LI-COR, Lincoln, NE, USA). The integrated density of the bands was quantified with ImageJ.

### Immunofluorescence

Frozen sections or cultured cells were fixed in 3.7% (v/v) paraformaldehyde. After permeabilization and blocking, the samples were immunostained with the primary antibody (Table 2) overnight at 4 °C, followed incubation with fluorescein-conjugated secondary antibody (Table 2). DAPI staining was used to reveal the nuclei of the cells.

### Animal experiments

Six 6–8 weeks old male C57BL/6 mice for each group were used for the experiments (n = 6). Shandong Eye Institute approved the study protocol, which was in accordance with the Association for Research in Vision and Ophthalmology (ARVO) Statement for the Use of Animals in Ophthalmic and Vision Research. Under general anesthesia, the entire corneal epithelium, including the limbal region, was scraped with algerbrush II corneal rust ring remover (Alger Co, Lago Vista, TX). Subsequently, 50 ng CNTF, or PBS as control, was injected subconjunctivally. The defect area of the corneal epithelium was evaluated after 24, 48 and 72 hours using 0.25% fluorescein sodium under a BQ900 slit lamp (Haag‐Streit, Bern, Switzerland). For Akt inhibition, normal mice were injected subconjunctivally with Akt inhibitor (0.65 μg/eye, Calbiochem, #124012, La Jolla, CA) 24 hours before the injection of CNTF and the scrape of corneal epithelium.

### Statistical analysis

All the experiments were carried out using at least three replicates and all the experiments were performed successfully at least three separate times. Quantitative data are presented as mean ± SD. Statistical analysis was performed using Student’s t-test when comparing two groups. One-way ANOVA and Bonferroni post hoc test were performed for comparison between more than two groups. Values of p < 0.05 were considered statistically significant.

## Additional Information

**How to cite this article**: Chen, J. *et al.* Ciliary Neurotrophic Factor Promotes the Migration of Corneal Epithelial Stem/progenitor Cells by Up-regulation of MMPs through the Phosphorylation of Akt. *Sci. Rep.* **6**, 25870; doi: 10.1038/srep25870 (2016).

## Supplementary Material

Supplementary Information

## Figures and Tables

**Table 1:** Primer sequences of mouse genes used for qPCR.

Genes	5′-3′	Primers	Production size (bp)
*Gapdh*	Forward	GCAAATTCAACGGCACAG	141
	Reverse	CACCAGTAGACTCCACGAC	
*Cntf*	Forward	ACCACAGGCATATTTCGTCA	144
	Reverse	GGTGGAAGGATAATGCCCTA	
*Mmp3*	Forward	CAGACTTGTCCCGTTTCCAT	173
	Reverse	GGTGCTGACTGCATCAAAGA	
*Mmp14*	Forward	GTACTACCGGTTCAATGAAGAAT	192
	Reverse	GGATACCCTGGCTCTACCTTC	

**Table 2:** Antibodies used for immunofluorescence staining and Western blot.

Antibody	Company	Code
CNTF	Abcam	ab10833
*p*-Akt	Cell signaling	#4060
MMP3	Abcam	ab53015
MMP14	Abcam	ab51074
Polyclonal Swine Anti-Rabbit Immunoglobulins/TRITC	Dako	R0156
Anti-rabbit IgG	Cell signaling	7074s
Donkey anti-goat IgG-CFL 488	Santa Cruz	sc362255
Donkey anti-goat IgG-HRP	Santa Cruz	Sc-2020
