# Supplementary material for: Ciliary Neurotrophic Factor Promotes the Migration of Corneal Epithelial Stem/progenitor Cells by Up-regulation of MMPs through the Phosphorylation of Akt
Source: Sci Rep. 2016 May 13;6:25870. doi: 10.1038/srep25870 (PMC4865747; doi:10.1038/srep25870)
Supplement: Supplementary Information [file srep25870-s1.pdf]

Ciliary Neurotrophic Factor Promotes the Migration of Corneal Epithelial Stem/progenitor Cells by Upregulation of MMPs through the Phosphorylation of Akt

Jialin Chen[1]; Peng Chen[2]; Ludvig J. Backman[1]; Qingjun Zhou[2, **]; Patrik Danielson[1,3, *]

[1] Department of Integrative Medical Biology, Anatomy, Umeå University, Umeå, Sweden

[2] State Key Laboratory Cultivation Base, Shandong Provincial Key Laboratory of Ophthalmology, Shandong Eye Institute, Shandong Academy of Medical Sciences, Qingdao, China

[3] Department of Clinical Sciences, Ophthalmology, Umeå University, Umeå, Sweden

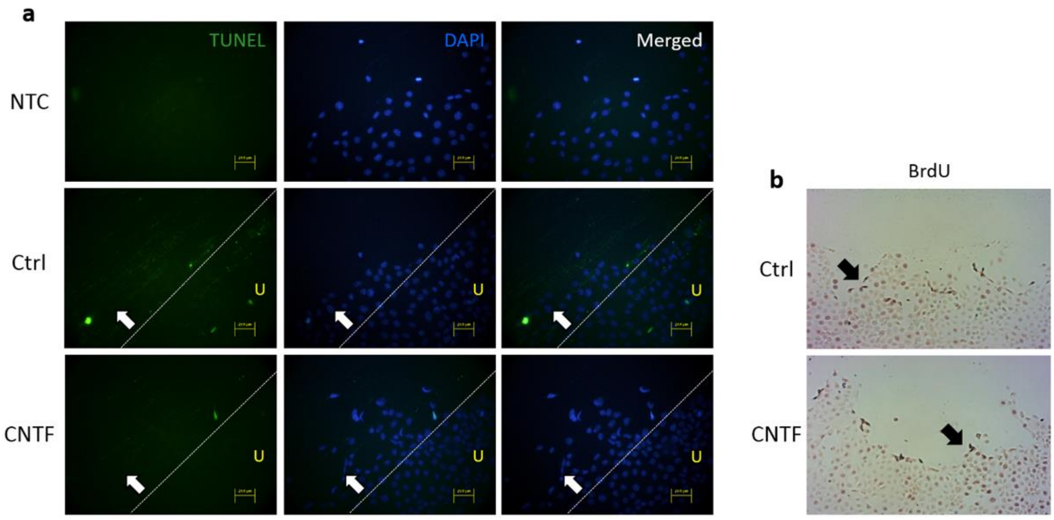

Supplementary Figure 1. Cell apoptosis and proliferation around the wound margins at 24h were compared between control group (Ctrl) and CNTF treated group *in vitro*. (a) Apoptotic cells were detected by the DeadEnd™ Fluorometric TUNEL System. The right column is the merged picture of left column and middle column. Dash line indicates the edge of scratch caused by the tip. 'U' indicates unwounded region. White arrows indicate the direction of cell migration. Negative control staining (NTC) in upper row. (b) BrdU staining were performed around the wound margins. Black arrows indicate cells labelled with BrdU.

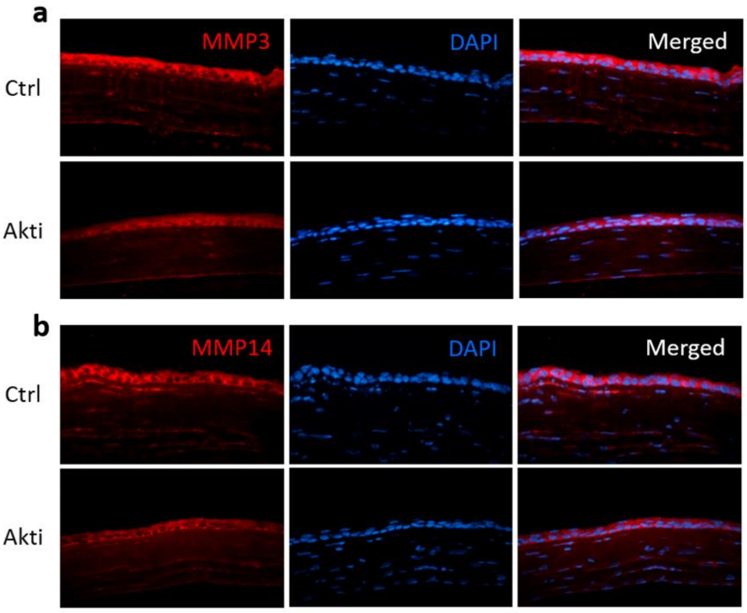

Supplementary Figure 2. Akt inhibitor treatment decreases the expression of MMP3 and MMP14 in corneal epithelium injured mouse. The expression of MMP3 (a) and MMP14 (b) was compared between Akt inhibitor (Akti) group and control group (Ctrl; injected with PBS) by immunofluorescence staining.

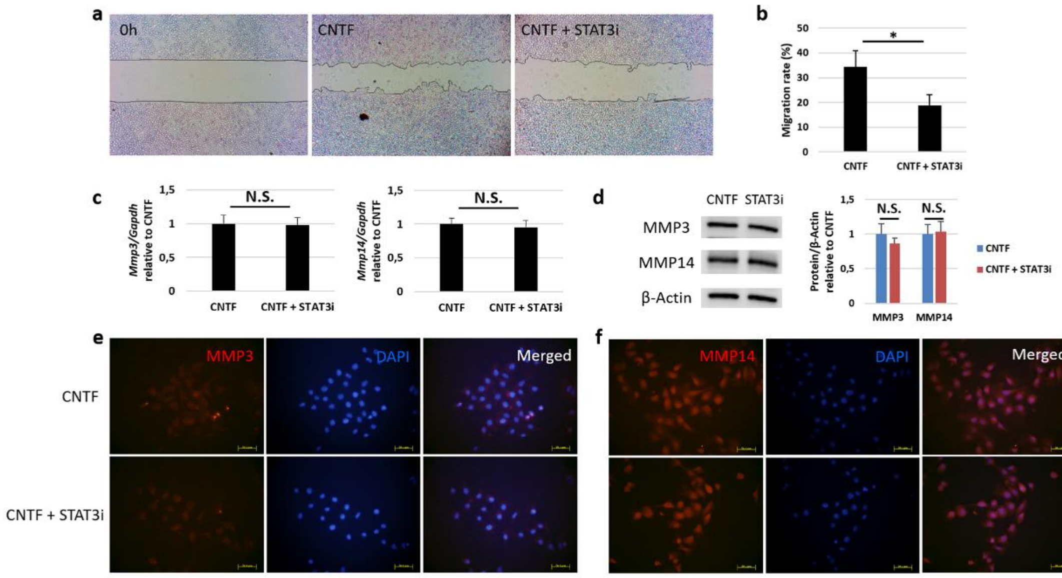

Supplementary Figure 3. STAT3 does not regulate the expression of MMP3 and MMP14 in mouse corneal epithelial stem/progenitor cells. (a) Confluent monolayer of TKE2 cells was scraped and treated with 10 ng/ml CNTF for 24 hours. For STAT3 inhibition, cultured TKE2 cells were pretreated with 100 µM STAT3 inhibitor for 30 minutes before CNTF treatment. (b) Image-Pro Plus 6.0 quantified the gap

area and the migration percent was calculated. (c) The mRNA level of *Mmp3* and *Mmp14* was evaluated by qPCR after STAT3 inhibitor treatment together with CNTF as compared to CNTF alone. (d) The protein level of MMP3 and MMP14 was evaluated by Western blot. (e and f) The expression of MMP3 and MMP14 after STAT3 inhibitor treatment was evaluated by immunofluorescence staining. The right column is the merged picture of left column and middle column. STAT3i, STAT3 inhibitor treatment. n = 3 per group. Statistical analysis was performed using Student's t-test. *Significant difference between two groups at $p < 0.05$. $^{N.S.}$No significant difference between two groups at $p \geq 0.05$.

Supplementary Methods

TUNEL staining

TUNEL staining was performed using DeadEnd™ Fluorometric TUNEL System (Promega, G3250). Confluent monolayer of TKE2 cells was scraped and treated with 10 ng/ml CNTF or not for 24 hours. Cells were mounted in DAPI-containing mounting medium after TUNEL. The fluorescein-12-dUTP-labeled DNA and DAPI was visualized by fluorescence microscopy.

BrdU labelling and detection

BrdU labelling and detection was performed using 5-Bromo-2'-deoxy-uridine Labeling and Detection Kit II (Roche, Basel, Switzerland). Confluent monolayer of TKE2 cells was scraped and treated with 10 ng/ml CNTF or not for 24 hours. Cells were incubated with BrdU labelling medium in a 5% CO_2 incubator at 37°C for 30 min. Next, cells were fixed and incubated with Anti-BrdU working solution for 30 min, followed by an Anti-mouse-Ig-AP solution incubation for 30 min. Subsequently, cells were incubated with freshly prepared Colour-substrate solution and evaluated in a light microscope.
